# Update on treatment and preventive interventions against COVID-19: an overview of potential pharmacological agents and vaccines

**DOI:** 10.1186/s43556-020-00017-w

**Published:** 2020-12-03

**Authors:** Yinan Xiao, Hanyue Xu, Wen Guo, Yunuo Zhao, Yuling Luo, Ming Wang, Zhiyao He, Zhenyu Ding, Jiyan Liu, Lei Deng, Fushen Sha, Xuelei Ma

**Affiliations:** 1 Department of Biotherapy, State Key Laboratory of Biotherapy, West China Hospital, Sichuan University, Chengdu, 610041 China; 2 West China School of Medicine, West China Hospital, Sichuan University, Chengdu, 610041 China; 3 West China Hospital, Sichuan University, Chengdu, 610041 China; 4 Infectious Diseases Center, West China Hospital, Sichuan University, Chengdu, 610041 China; 5 Department of Pharmacy, State Key Laboratory of Biotherapy and Cancer Center, National Clinical Research Center for Geriatrics, West China Hospital, Sichuan University, and Collaborative Innovation Center of Biotherapy, Chengdu, 610041 China; 6 Jacobi Medical Center, Albert Einstein College of Medicine, Bronx, New York, 10465 USA; 7 Department of Internal Medicine, State University of New York, Downstate Medical Center, Brooklyn, New York, 11203 USA

**Keywords:** SARS-CoV-2, COVID-19, Antiviral drugs, Immunotherapy, Vaccines

## Abstract

The outbreak of coronavirus disease 2019 (COVID-19) triggered by the new member of the coronaviridae family, severe acute respiratory syndrome coronavirus 2 (SARS-CoV-2), has created an unprecedented challenge for global health. In addition to mild to moderate clinical manifestations such as fever, cough, and fatigue, severe cases often developed lethal complications including acute respiratory distress syndrome (ARDS) and acute lung injury. Given the alarming rate of infection and increasing trend of mortality, the development of underlying therapeutic and preventive treatment, as well as the verification of its effectiveness, are the top priorities. Current research mainly referred to and evaluated the application of the empirical treatment based on two precedents, severe acute respiratory syndrome (SARS) and Middle East respiratory syndrome (MERS), including antiviral drugs targeting different stages of virus replication, immunotherapy modulating the overactivated inflammation response, and other therapies such as herbal medicine and mesenchymal stem cells. Besides, the ongoing development of inventing prophylactic interventions such as various vaccines by companies and institutions worldwide is crucial to decline morbidity and mortality. This review mainly focused on promising candidates for the treatment of COVID-19 and collected recently updated evidence relevant to its feasibility in clinical practice in the near future.

## Introduction

Coronavirus disease 2019 (COVID-19) is a severe acute respiratory syndrome that has infected more than 23,300,000 patients and caused 806,410 deaths from 216 countries and territories so far. The pathogen of COVID-19 is severe acute respiratory syndrome coronavirus 2 (SARS-CoV-2), a new member of the coronaviridae family that also includes severe acute respiratory syndrome coronavirus (SARS-CoV) and Middle East respiratory syndrome coronavirus (MERS-CoV) [1–3]. Bats, the natural reservoirs of SARS-CoV and MERS-CoV, might also be the source of COVID-19 due to the similarity of RaTG13 from the short RNA-dependent RNA polymerase (RdRp) region between bat coronavirus and SARS-CoV-2 [1, 4, 5]. The main mode of transmission is airborne, contact transmission and respiratory droplets and the median incubation period from exposure to onset for COVID-19 was about 3.0 days [6].

The majority of confirmed cases are between 30 and 79 years of age and that patients older than 60 tend to develop more serious symptoms or even die [7, 8]. Approximately 25.2–50.5% of patients with SARS-CoV-2 infection have one or more underlying diseases, including hypertension, diabetes, chronic obstructive pulmonary disease, cardiovascular disease, and malignancy [9, 10]. The clinical manifestations of patients with SARS-CoV-2 infection range from mild non-specific symptoms to severe pneumonia with organ function damage. The main clinical symptoms of COVID-19 are fever (83–98%), cough (59–82%), shortness of breath (19–55%), weakness (38.1–69.6%), sputum production (28.2–56.5%), headache (6.5–33.9%) and muscle aches (11–44%), which are similar to severe acute respiratory syndrome (SARS) and Middle East respiratory syndrome (MERS) [11].

For COVID-19, chest computed tomography (CT) plays a very important role in detecting infected individuals, with imaging showing mainly ground-glass opacity, interstitial abnormalities, patchy shadowing, crazy-paving pattern and septal thickening [12–14]. Therefore, the CT images of COVID-19 change in a variety of forms rapidly [15]. Besides, the changing levels of C-reactive protein (CRP), erythrocyte sedimentation rate, serum ferritin and interleukin-6 (IL-6), d-dimer, lactate dehydrogenase and creatine kinase might also indicate the disease progression [16].

Confirmation of SARS-CoV-2 infection mainly relies on the positive results of high-throughput sequencing or real-time reverse transcriptase-polymerase chain reaction (RT-PCR) test results [17]. Additionally, chest CT with its high sensitivity to COVID-19, has been given more value in the diagnosis [18].

The current treatment of COVID-19 depends on existing antiviral drugs and immunotherapy [19]. The mechanism of antiviral drugs is targeting various stages of the viral invasion pathway including virus recognition, fusion, entry and genome proliferation. Currently the main targets are the angiotensin-converting enzyme 2 (ACE2) receptor and the transmembrane protease/ serine subfamily member 2 (TMPRSS2) and common types of drugs are protease inhibitors, RNA polymerase inhibitors and interferons [20]. SARS-CoV-2 induces a hyper-inflammatory state characterized by an excessive immune response and cytokine dysregulation, which eventually leads to cytokine storms and fatal complications [21]. Thus, in addition to antiviral drugs and symptomatic treatment, immunomodulatory therapy is another critical measure. Common treatment options include corticosteroids, anti-cytokine drugs, Janus kinase (JAK) inhibitors, chloroquine (CQ), hydroxychloroquine (HCQ), convalescent plasma, Intravenous immunoglobulin (IVIG) and interferon (IFN). In addition to these two broad categories of treatment options, stem cell therapy and traditional herbal treatments could also be promising medication [22, 23]. For the prevention of COVID-19, a large number of vaccines are already in the development process, mainly including mRNA vaccine, DNA vaccine, recombinant vaccine Ad5-nCoV [24, 25].

In this review we collected updated evidence regarding the usage of various therapies for COVID-19 in clinical practice and its feasibility, hoping to offer helpful instructions for clinical management and strategies.

## Introduction of SARS-CoV-2

**Fig. 1:**
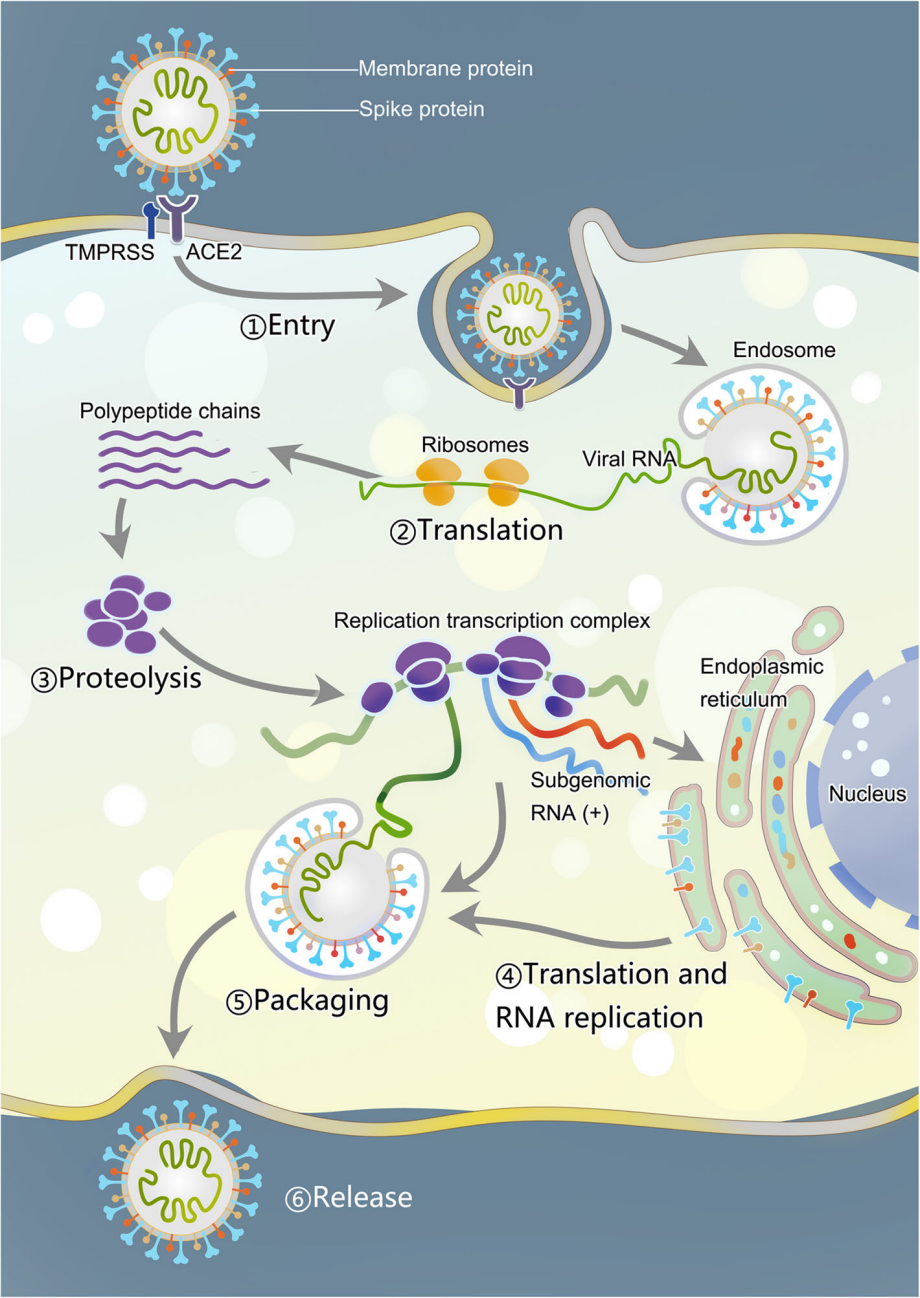
The membrane fusion, replication, packaging and release of SARS-Cov-2. SARS-CoV-2 has four structural proteins, including spike (S), membrane (M), envelope (E), nucleocapsid (N) proteins. ①The entry of coronavirus into host cells is mediated by the S glycoprotein, which can be activated by transmembrane protease/ serine subfamily member 2 (TMPRSS2). ②After the entry of coronaviral genome, viral genomic RNA will start to replicate and synthesize polypeptide chains. ③These polypeptide chains later forms proteolysis which constitutes replication transcription complex to assist the synthesis of other viral structural proteins ④Following the synthesis of genomic and sub-genomic RNA replication, the S, E, and M proteins are translated and then they are sequentially transported along the secretory pathway into the endoplasmic reticulum ⑤Then the proteins are modified and packaged in the endoplasmic reticulum-Golgi intermediate compartment. ⑥Inside the compartment, the viral genome enveloped by the N protein will bud into the membrane, thus forming and releasing a mature virus

SARS-CoV-2 is tightly associated with SARS-CoV, both originating from bat [26–29]. For SARS-CoV, the intermediate hosts for zoonotic transmission of SARS-CoV between bats and humans are palm civets and raccoon dogs, while for SARS-CoV-2, the intermediate hosts have not been identified yet [30, 31]. Many pathogenic zoonotic pathogens belong to the b-coronavirus genus, including viruses with high pathogenic rate: SARS-CoV, MERS-CoV, and SARS-CoV-2 and four low-pathogenicity coronaviruses: HCoV-OC43, HCoV-HKU1, HCoV-NL63, and HCoV-229E [32]. Coronaviruses get their name because their outer membrane looks like a crown under an electron microscope. The main pathogenesis of SARS-COV is the direct infection of macrophages and T cells, and SARS-COV-2 may also be pathogenic through infection of immune cells [33]. The mechanisms of SARS-COV-2 injury have been proposed, including: (1) infecting target cells expressing ACE-2, such as immune cells; (2) inhibiting IFN response and promoting virus replication; (3) increasing the activation of neutrophils and macrophages and the release of proinflammatory cytokines, leading lung injury; and (4) activating specific Th1/Th17 and B cells, leading to a series of inflammatory responses associated with SARS-CoV-2 antibodies [34]. The structure of SARS-CoV-2 and its reproduction in the host cells are described as follows (Fig. 1).

### Membrane fusion and virus invasion

Spike (S) glycoprotein, a 150 kDa highly N-glycosylated protein, protrudes on the viral surface as homotrimers and plays a crucial in the entry of coronavirus into host cells [35]. S protein is composed of two functional subunits: S1 subunit contains N-terminal domain (NTD) and C-terminal domain (CTD), and S2 subunit is transmembrane and has a short cytoplasmic domain. Before fusion, S1 and S2 are noncovalently bound in many CoVs [36–43]. The primary function of S1 subunit is to bind to host cell receptors, while the main function of S2 subunit is to mediate the fusion of virus and cellular membrane. In addition to receptor-binding, S1 subunit also plays a role in stabilizing the prefusion state of membrane-anchored S2 subunit [39, 43–46]. During the process of membrane fusion between virus and susceptible cells, S protein needs to be activated by S2’ site, which is located upstream of fusion peptide and forms irreversible conformational changes through the host proteases [36, 41, 42, 47, 48]. Then, a fusion peptide will be inserted into the host cell membrane, and two heptad repeats in S2 will join together to form an antiparallel six-helix bundle [37]. Thus, the entry of coronavirus into susceptible cells involves two main processes, proteolytic activation and receptor binding. Each kind of coronavirus has a specific S1 subunit and a corresponding invasion receptor. For example, the structure involved in the surface recognition of MERs-CoV S is domain A, which can recognize the non-acetylated sialoside attached receptor and facilitate the binding of domain B (SB) and the entrance receptor dipeptidyl peptidase 4 [49–53]. For SARS-CoV and several SARS-related coronaviruses, the entry of the virus into the target cell is mediated by SB binding to the receptor of ACE2 [26, 46, 54, 55].

The S trimer is decorated with N-linked glycan, which guarantees proper folding, modulates the interaction with host proteases and neutralizes Abs [56–58]. Due to S trimer exists on the surface of virus and can mediate virus invasion, it is an important target for drug design. Previous studies used human-neutralizing Abs from patients infected with SARS-CoV [59] or MERS-CoV [60] and S from SARS-CoV and MERS-CoV to explore the mechanisms by which SB attaches to host receptors [57]. Human ACE2 (hACE2) is a receptor, which highly expresses on intestinal cells and lung cells. It has a comparable affinity to SARS-CoV SB and SARS-CoV-2 SB, which both use the C-terminal domain to interact with hACE2 [61]. The structure of SARS-COV-2-CTD is similar to that of SARS-COV, and the sequence consistency is up to 73.9%. There are many similar binding sites between SARS-COV-2 and SARS-COV in their binding to hACE2, indicating that CoV has evolved to bind to hACE2 in the “hotspot” region [61]. However, SARS-COV-2 S protein binds to hACE2 more closely than SARS-COV with more chemical bonds and a larger buried surface area [61, 62].

### Replication, assembly and release

After the entry of the coronaviral genome, the viral genomic RNA begins to replicate. The replication of viral RNA depends on the materials of the host cells. During the process of replication, the RNA polymerase interacts with the lead sequence of the viral genomic RNA to produce a nested set of mRNAs with common 3′ ends. Two or three proteases will be encoded to cleave the replicase polyproteins. During the process of RNA synthesis, a number of the nonstructural proteins gather to the replicase-transcriptase complex, creating a suitable environment and assisting in the synthesis of negative-strand intermediates. Sub-genomic RNA located downstream of replicase polyproteins is the basis of structure and assists gene expression [63]. Homologous and non-homologous recombination is a feature of coronavirus. It is associated with the strand switching ability of the RdRp and is the basis for virus evolution [64, 65]. With the synthesis of genomic and sub-genomic RNA replication, S, E, and M proteins are translated and then modified and packaged in the endoplasmic reticulum and endoplasmic reticulum-Golgi intermediate compartment [66, 67]. Inside the compartment, the viral genome, which is wrapped in the N protein, buds into the membrane and forms a mature virus [68].

## Antiviral drugs for COVID-19

### Protease inhibitors

#### Lopinavir/ritonavir

Lopinavir (LPV) and ritonavir (RTV) are two protease inhibitors with related structure. LPV is an antiretroviral drug that prevents the proliferation of the virus and is widely used in treating human immunodeficiency virus (HIV) infection. LPV can specifically inhibit HIV-1 protease, thereby inhibiting HIV-1 proliferation in host cells and blocking HIV-1 infection. The pesticide effect of LPV can be enhanced by RTV, which presents no effect when used alone against SARS-CoV-2. RTV promotes the efficacy of LPV by decreasing the hepatic metabolism of LPV. The recommended dose of LPV/r, 400 mg/100 mg twice daily, is based solely on the plasma concentration of LPV, under which LPV can suppress about half of viral replication in immune cells effectively [69]. Drug-drug interaction (DDI) is the main problem of Lopinavir/ritonavir (LPV/r) because they are both substrates and inhibitors of many drug-metabolizing enzymes, like cytochrome P450 3A4, and drug efflux transporters, like P-glycoprotein. Thus, they can be toxic when used with many other medicines [70–72].

Many studies have evaluated the efficacy of LPV/r. The performance of LPV/r in tissue culture models is controversial. A study that screened a library of 348 Food and Drug Administration (FDA)-approved drugs to find drugs with anti-MERS-CoV activity found LPV can inhibit MERS-CoV replication with low-micromolar concentration. However, another study that selected LPV from a chemical library of 1280 kinds of drugs by chemical methods had unsatisfactory in vitro results [73, 74]. In animal studies, the MERS model of common marmoset treated with LPV/r showed positive results, including less weight loss, viral titers, and better clinical scores and disease prognosis [75]. LPV alone had 50% effective concentration (EC50) ranged from 6.6 to 17.1 mM when used against SARS-CoV, MERS-CoV, and hCoV-229E in vitro [74]. LPV/r was effective for both patients and tissue effected by SARS-CoV. Forty-one patients with SARS received both LPV/r and RBV for 3 weeks and had significantly less adverse outcomes compared with the historical controls [76]. Furthermore, both a non-randomized open-label trial and a randomized trial found LPV/r can improve the clinical outcomes of patients with SARS-CoV [76–78]. There are 59 ongoing studies and 5 completed studies exploring the application of LPV/r in treating COVID-19 (NCT04358614, NCT04343768, NCT04276688, NCT04379245, NCT04374071). Several COVID-19 case series reported ambiguous results, in which some patients got a lower viral load and sooner recovery, while others deteriorated [2, 79–81]. Moreover, a randomized, controlled, clinical trial (Chinese Clinical Trial Register number, ChiCTR2000029308) with 199 patients involved found no significant benefit from LPV/r treatment [3]. The poor performance of LPV/r in SARS-CoV-2 can be because that the SARS-CoV-2 counterpart (3CLpro) is dissimilar to the HIV aspartic protease [82]. Increasing combination therapies of LPV/r were explored with positive results, including LPV/r with arbidol, ribavirin and IFN-β [83–85]. However, there were studies indicated that the combination of LPV/r and IFN-β did not have better potency than using IFN-β alone [86]. In terms of side effects, LPV/r can increase the occurrence of liver injury, but reduce the occurrence rate of overall death, acute respiratory distress syndrome and nosocomial infection. Due to the lack of large-scale studies about the pesticide effect of LPV/r, many clinicians still advocate the use of LPV/r as it is relatively safe and convenient for deployment [87].

#### Camostat mesylate

Camostat mesylate (CM) is a protease inhibitor developed in Japan, in the 1980s, and firstly used for chronic pancreatitis and then postoperative reflux esophagitis with an oral dose of 600 and 300 mg/day, respectively [88–91]. It is now regarded as a potential drug for curing COVID-19, since S protein driven viruses need TMPRSS2 to active S protein to ensure their entry, and CM can inhibit this process [92–94]. Its efficacy has been approved in cellular level, as it can effectively block the spare of SARS-CoV and HCoV-NL63 into the HeLa cells and the entry of SARS-CoV-2 into the human lung Calu-3 cells [95, 96]. In a SARS-CoV mouse model experiment, CM effectively protected 60% mouse from death [91]. In this study, the amount of CM required for humans was 2.14 mg/kg after converting the human and mouse weights. This dose was considered to be under the safe dose. The plasma half-life of CM is 100 min, and CM almost completely disappears in plasma after 4-5 h. Therefore, taking 600 mg CM daily may be an effective way to control SARS-COV-2 infection. CM is very safe to use in Japan, where 100,000 people take it each year and few adverse events have been reported [97]. Meanwhile, low cost is the main advantage of CM, taking only 0.1–0.4 USD for a 100 mg tablet, being especially beneficial to the patients with low-income. Therefore, CM is likely to become an effective treatment for COVID-19 after more clinical trials.

### RNA polymerase inhibitor

#### Remdesivir

Remdesivir (RDV) is a 1′-cyano-substituted adenosine C-nucleotide ribose analogue, developed by Gilead Sciences to cope with Ebola and related viruses. It can inhibit the proliferation of virus by targeting the RNA polymerase and its antiviral activity depends on the active triphosphate metabolite [98, 99]. Compared with other members of 1′-cyano group, whose interference with viral transcription is slowed down by the slow first phosphorylation kinetics, RDV can avoid this barrier as it is a isomeric compound of the 2-ethylbutyl l-alaninate phosphoramidate prodrug [100]. When RDV enters the virus, its triphosphate form, which is similar to ATP, will be used as a substrate for RdRp in several viruses and interfere the virus proliferation [101–103]. Some studies also reported that in the fight against Ebola, Nipah and respiratory syncytial viruses, RDV mainly delays the termination of new viral RNA strands [101, 103–105]. Moreover, there are many other pathogenic RNA viruses (including Filoviridae, Paramyxoviridae, Pneumoviridae, and Orthocoronavirinae) that can be inhibited by RDV in vitro, suggesting that it has a wide range of potential medical applications [106].

Though RDV had no effect in curing Ebola, it was therapeutic for MERS and SARS both in tissue culture and animal studies [10, 106, 107]. In the cultures of primary human lung epithelial cells, RDV can effectively suppress the proliferation of SARS-CoV and MERS-CoV, with EC50 of 0.07 mM [106]. In the mouse model effected by SARS-CoV, RDV can decrease pulmonary viral load and improve both clinical symptoms and respiratory function effectively. On the fourth or fifth day after infection, the virus titer was reduced by two orders of magnitude [108]. In addition, RDV presented high intracellular concentration (> 10 mM) in a rhesus monkey Ebola model. Daily intravenous injection of 10 mg /kg RDV could improve the clinical symptoms and pathophysiological indicators. Meanwhile, RDV can prevent and treat the rhesus macaque model with MERS-CoV infection. Lower MERS-CoV replication level and fewer gross and histologic lung lesions existed in the test group with RDV [103, 109]. For mice affected by MERS, RDV was proven to be superior to the combination of LPV/r and IFN-β, and could reduce MERS-CoV replication, acute lung injury, and improve pulmonary function [86]. In the fight against COVID-19, RDV was widely used in the USA and Europe, known as compassionate use. The first COVID-19 case in the USA was successfully cured by RDV on day 7 of hospitalization. Furthermore, it is difficult for CoV to develop resistance to RDV. In a previous study, which introduced murine hepatitis virus resistance mutations into SARS-CoV, though SARS-CoV gained drug resistance, the pathogenesis of SARS-CoV also decreased [110]. Nevertheless, recent studies reported that there were still some uncertain treatment effects and side effects (like vomiting, nausea, rectal hemorrhage, and hepatic toxicity) of RDV. Thus, more studies are in demand for evaluating the safety of RDV. At present, nine Phase 3 human trials (NCT04315948, NCT04321616, NCT04280705, NCT04292730, NCT04292899, NCT04359095, NCT04395170, NCT04361461, NCT04349410) and three Phase 2 human trials (NCT04386447, NCT04330690, NCT04373044) are in progress about using RDV for moderate to severe adult SARS-CoV-2 cases and may have preliminary results soon.

#### Ribavirin

Ribavirin (RBV) is a broad-acting antiviral drug and can suppress the viral proliferation by multiple mechanisms. First designed for children with respiratory syncytial virus in the 1980s, it is now also used for viral hemorrhagic fever and in combination with IFN for hepatitis C [85]. It can act as a cytostatic agent to reduce the synthesis of DNA, RNA and proteins in cells [111]. RBV is clinically administered as the nucleoside, which can be converted to ribavirin monophosphate (RMP) by adenosine kinase. The RMP will subsequently be phosphorylated into di- and tri-phosphorylated nucleotides, which is generally the dominating metabolite [112]. In vitro, RBV has a relatively short half-life in cultured fibroblasts and lymphoblasts and a longer half-life in erythrocytes, which is the cause of its side effect, reversible hemolytic anemia [112]. In addition to interfering with polymerase, RBV also promotes RNA degradation by interfering with RNA capping, decreases the stabilization of viral RNA by inhibiting the generation of guanosine, reduces the fidelity of viral nucleic acid replication by introducing random mutations and indirectly fights against virus by mediating the immune system [113, 114]. Meanwhile, as an immunomodulator, RBV promotes the transformation of T-helper cell phenotype from type 2 to type 1 [114]. Cellular immunity is activated during the T-helper type 1 response and is associated with the expression of interleukin-2 (IL-2), interferon-gamma, and tumor necrosis factor-alpha [115, 116]. In the hepatitis C virus infection, a T-helper 2 response may lead to the development of chronic disease [117]. Thus, the inhibition of a Type-2 response and promotion of a Type-1 response of RBV can help combat the virus. The pharmacokinetics and bioavailability of RBV in human have been thoroughly studied, and the EC50 of RBV against COVID-19 is much higher than the semi-maximum inhibition concentration of RBV against dengue virus [118, 119]. In addition, adequate experience in clinical use, easy access to get, relatively affordable price are also the reasons for choosing this drug [120].

In the model of rhesus macaque effected by MERS-CoV, the group treated by RBV with IFN-α2b 8 h after inoculation did not develop breathing abnormalities, no or very mild pneumonia sign in radiographic evidence, and low levels of systemic and local proinflammatory markers [121]. However, in a study that included 349 patients under the infection of MERs-CoV, combined use of RBV and IFN on average 2 days after admission to the intensive care unit did not reduce 90-day mortality or accelerate MERS-CoV RNA clearance [122]. In 2003, the results of RBV combined with corticosteroids and/or IFN in the treatment of SARS-COV patients were not optimistic, and many adverse reactions occurred in patients, in particular increased hemolysis and transaminase [123, 124]. Although RBV shows inhibitory activity at viral load, it is not practical to treat SARS-COV with RBV because this effect is only available at high concentrations (0.5–5 mg/mL), which have been shown to have cytotoxic effects on VeroE6 cells [125, 126]. In addition, the dose of RBV required to treat SARS patients (1.2–2.4 g, three times a day) was excessively toxic to patients [127]. Compared with RDV, the in vitro dose (EC50 109.5 mM) of RBV for SARS-CoV-2 was 100 times higher than that of RDV [119]. According to the results of cell experiments, the dosage of RBV can be reduced when combined with IFN [128, 129]. A multicenter, randomized study (NCT04276688) compared the combination use of RBV, LPV/r and IFN-β and LPV/r alone. The therapeutic effect of the former was superior to that of the latter [130]. The side effects of RBV are relatively strong, especially hemolytic anemia [123, 131, 132]. A retrospective study in Canada suggested that RBV might not be clinically effective, but could increase the incidence of adverse events and lead to early drug withdrawal [123]. In addition, the US FDA has made it clear that RBV has strict indications and is not recommended for the treatment of influenza [133]. Therefore, there is insufficient evidence to prove the effect of RBV on SARS-CoV-2 based on the current research results [123, 134].

#### Interferon-α

IFN-α is a broad-spectrum antiviral drug that has been used to treat viral hepatitis and block SARS-CoV virus replication. IFN type-I response and downstream cascade play important roles in innate immune responses, mediating the effective activation of adaptive immune response [135, 136]. IFN fights against the virus by stimulating multiple immune pathways that include four major factors: 2′-5′-oligoadenylate synthetase (OAS) protein, ribonuclease L (RNase L), double-stranded RNA-dependent protein kinase (PKR), Mx proteins and RNA-specific adenosine deaminase (ADAR)-1. OAS protein cooperate with RNase L and can be activated by dsRNA to polymerize ATP into 2′-5′-linked oligoadenylates (2–5(A) with various length and degrade single-stranded RNA [137]. This antiviral ability of the 2–5(A) synthetase system has been reported in HCV, vaccinia and HIV [138]. In addition, OAS can induce apoptosis by blocking the effects of B-cell leukemia/lymphoma (Bcl)-2 and Bcl-xl [139]. PKR can be induced by IFN and is associated with viral dsRNA. Upon activation, PKB inhibits protein synthesis by phosphorylating eukaryotic initiation factor 2α subunits and interferes with a variety of signal transcription pathways, including STAT1 and nuclear factor-KappaB (NF-kB). The activity of IFN is also mediated by PKR, because the mutation or suppression of PKR can influence the anti-EMCV action of type I IFNs [140, 141]. The antiviral ability of Mx proteins does not require collaboration with any other IFN-induced cellular proteins. Mx proteins can interfere with the synthesis of viral RNA and block the transport of viral nucleocapsid by binding to viral proteins and protein-protein interactions, and thus has a wide range of antiviral activity [142–144]. The IFN-inducible ADAR family leads to hypermutability in dsRNA of many viruses because it can substitute adenosines (A) with inosines (I) [145, 146]. This conversion decreases the stability of dsRNA and inhibits the replication of viruses, including measles, polyomavirus, vesicular stomatitis, and hepatitis D virus [147, 148].

At present, IFN-α subtypes are approved for clinical use, including IFN-α2b, IFN-α2a, and IFN-α1b, and IFN-α2b has relatively higher activity [149]. In a SARS-CoV-2 Vero cell study, IFN-α and IFN-β effectively reduced viral titers to the concentration of 1.35 IU/ml and 0.76 IU/ml, indicating SARS-CoV-2 infection can be inhibited by IFN in cell culture [135]. Although the efficacy of IFN-α has been proved in the clinical trials of MERS infection, a small cohort study found that IFN-α was useful only in the early and intermediate stage of MERS. Several clinical trials are currently under way to evaluate the efficacy of Type-I IFN alone (NCT04293887, NCT04320238, ChiCTR2000029989) or in combination (NCT04254874, NCT04273763, NCT04276688, NCT04343768, NCT04350671) against COVID-19. In a double-blind clinical trial, although low-dose oral IFN failed to cure acute respiratory disease, it did reduce the severity of symptoms and benefit subgroups of patients [150]. In a retrospective single-center study involving 94 confirmed COVID-19 patients, therapeutic regimens of IFN-α + LPV/r and IFN-α + LPV/r + RBV were found to be beneficial for reducing IL-6 and C-reactive protein levels in COVID-19 patients [151]. A retrospective study of COVID-19 patients treated alone or in combination with IFN-α2b and arbidol found similar results. The results showed a decrease in the level of the upper respiratory tract virus and a decrease in the duration of elevated levels of inflammatory markers IL-6 and CRP in the blood. Despite the positive results, the absence of a control group and the baseline mismatch between the groups reduced the reliability of the study results [152]. With regard to IFN for prevention, a prospective study (NCT04320238) recruited 2944 health care workers and classified them into low-risk and high-risk groups based on whether they had direct contact with COVID-19 patients. IFN-α nasal drops were administered to the low-risk group, and IFN-α nasal drops and thymosin-a1 were administered to the high-risk group. In terms of side effects, the main adverse reactions after subcutaneous and intramuscular injection of IFN-α are influenza-like symptoms, bone marrow suppression and mental disorders [153]. Inhalation may reduce systemic side effects but can still cause bronchospasm and prevent normal exhalation. In addition, the dose required by inhalation is relatively large, and IFN-α dose less than 18 × 10^6^ IU/ day is difficult to enter systemic circulation [154]. In completed studies, IFN-α was protective against SARS-CoV-2 and was often used in combination with other antiviral drugs to cure SARS-CoV-2 infection, suggesting it as a potential drug for COVID-19.

### Summary of antiviral drugs

Based on the mechanism of virus invasion, many antiviral drugs have been developed. Different drugs target different processes, including virus recognition, fusion, entry and genomic proliferation to prevent the spread of the virus. ACE2 receptor and protease TMPRSS2 play important roles in the virus recognition and fusion prior to virus entry into host cells. The protease inhibitors LPV/ R and CM, which have been most clinically studied on COVID-19, inhibit the activation of S protein by inhibiting the protease TMPRSS2. After entry, viral genome proliferation is inhibited by nucleotide analogues, like RDV and RBV that target RNA polymerase. In addition, since the invasion and replication of SARS-CoV-2 can induce the innate and adaptive immune responses, immunomodulators such as the IFN family can help active more effective immune cells to fight against SARS-CoV-2. Although the efficacy of these drugs has been abundantly studied in previous widely epidemic virus, MERS and SARS-CoV by cell culture, animal experiments and clinical trials, the best option for treating COVID-19 remains unclear. Therefore, more random and multicentral trails are required for better COVID-19 treatment. Besides the efficacy, side effects, cost and availability are also key considerations when choosing a drug. In order to promote the clinical use of these potential drugs, the World Health Organization and the European Union recently initiated clinical trials to test the efficacy of LPV/r plus IFN-β, RDV, chloroquine and hydroxychloroquine in COVID-19 patients worldwide in the SOLIDARITY Trial (NCT04321616) and the DisCoVeRy Trial (NCT04315948), and promising results may come out soon.

## Immunotherapy

### Corticosteroids

The clinical efficacy of corticosteroids, a potent regulator in the development of autoimmune disease and inflammatory process, has been verified in various indications. Both the classical pathway mediated by the receptor of corticosteroids that directly combined with glucocorticoid response elements to control certain gene expression and the transcription-independent non-classical pathway that leads to rapid signaling and persistent hormone effect underlies the physiological action of corticosteroids [155]. The advantage of inhibiting the exuberant immune response as well as the progression of pulmonary fibrosis theoretically allows the application in COVID-19 [156], especially for the severe case with acute respiratory distress syndrome (ARDS), the common lethal complication of viral infections in respiratory system [157, 158].

During the SARS and MERS outbreak, corticosteroids have been routinely used to forestall excessive lung damage and respiratory distress caused by hyperactive immune response [156, 157]. However, studies on corticosteroid treatment fail to provide supporting proof for its appropriate use but uncover underlying risks such as the low efficacy of virus clearance in patients with MERS infection [134, 159]. Observational studies of patients with influenza-associated pneumonia treated with corticosteroids also reported a higher risk of motility and hospital-acquired infection [160–162].

Evidence regarding the effectiveness of corticosteroids also disagreed on their clinical use. Notably, one study found that among 84 patients who developed ARDS out of 201 patients with COVID-19, methylprednisolone treatment significantly decreased the motility by 62% [163]. Another meta-analysis also attested to such therapeutic benefits in severe patients with ARDS but failed to show a similar conclusion in patients without ARDS [164]. Empiric therapy of corticosteroids in COVID-19 patients without conclusive evidence sometimes failed to exert positive effects on the disease progression. One study analyzed clinical information of 31 patients with COVID-19 and found that corticosteroids did not exert a significant effect on virus clearance and duration of hospitalization [165]. However, two retrospective studies of symptomatic patients suggested the association between corticosteroid treatment and prolonged viral RNA shedding as well as high risk of death [166]. Furthermore, a meta-analysis including 5270 patients indicated that corticosteroid treatment led to longer hospitalization days and higher mortality [79]. Recently, another meta-analysis of patients with SARS-CoV, MERS-CoV, and SARS-CoV-2 further demonstrated the increased use of mechanical ventilation under corticosteroid therapy [167]. Although the latest guidance (on the clinical management of COVID-19) from WHO recommended corticosteroid therapy in the severe and critical case, it also warned against regular administration of corticosteroids except for specific indications such as chronic obstructive pulmonary disease exacerbation or septic shock [168]. Consequently, until now, even though we have primarily recognized the efficacy and risk of corticosteroids in COVID-19 treatment, further research is needed to guide the appropriate use in clinical practice for its existing uncertainties [169].

In conclusion, the efficacy of corticosteroids in COVID-19 treatment is a double-edged sword. In clinical practice, the adverse effects of immunomodulation, for instance, immunosuppression and secondary infection, provoke different opinions on the usage of corticosteroid treatment. Now, subject to the limited sample numbers, short follow-up duration, and different administration plans, clinical evidence does not suffice to yield convincing conclusions, and therefore necessitates the urgent verification of the reasonable use of corticosteroid in large scale randomized controlled trials (RCTs). Two clinical trials (NCT04374071/NCT04273321) on the therapeutic effects of methylprednisolone on COVID-19 have been completed, the result of which has not been published so far. Ongoing clinical trials may provide us with more insights on the indications of corticosteroids in COVID-19 patients, the precise control of the administration time, interval, and appropriate dosage to avoid the potential risk but mitigate the suffering of patients as much as possible.

### Anti-cytokine interventions

Anti-cytokine interventions or other immunomodulatory agents might contribute to mitigating the overactivated host immune response induced by highly active proinflammatory cytokines [170] and further prevent detrimental complications such as ARDS and multiorgan dysfunction in COVID-19 patient [171]. Delayed type I interferon response, responsible for the initiation and amplification of cytokine storm in the COVID-19, activated extensive IFN-stimulated gene expression and recruited various innate immunocytes while various cytokines released further dampen T cell response that was crucial to the virus clearance [172]. Since the increasing level of IL-6, a pivotal role in the cytokine storm, is highly relevant to respiratory distress or poor outcomes [80, 173, 174], suppressing IL-6 and its receptors could alleviate the disease progression and promote the prognosis. Potential mechanism includes maintaining perforin expression at functional level [175], preventing overactivated Th17 cells [176], inhibiting NF-κB [177] and trans-signaling induced by membrane-bound gp130 which the soluble IL-6-IL-6R interact with [178].

Tocilizumab, a recombinant humanized monoclonal antibody targeting at both soluble and membrane-bound IL-6 receptors, is widely applicable to treating various immune-related disorders such as rheumatoid arthritis, adult-onset Still’s disease, juvenile idiopathic arthritis, giant cell arteritis, and cytokine release syndrome, a severe complication in chimeric antigen receptor T cell therapy [179]. A systematic review including both early case reports and retrospective studies of Tocilizumab revealed that it could be a potential promising treatment based on the preliminary result of improved clinical course [180]. More recent studies further attested to the potential benefits of Tocilizumab for COVID-19 patients [181–185]. A single-center prospective study of 100 patients with COVID-19 pneumonia and ARDS requiring respiratory support demonstrated that 77% of the patients showed great clinical improvement in respiratory function and imageological characteristics of the lung [186]. Another systematic review including 11 case reports of COVID-19 patients indicated that tocilizumab could effectively inhibit the hyper-inflammatory state by downregulating the level of IL-6 and CRP level [187]. In addition to the verification of the clinic effectiveness, a single-center retrospective study compared severe patients undergoing anti-cytokine treatments who later received ventilation with those who did not and suggested that the optimal administration timing for anti-cytokine therapy might be before ventilation in intensive care units (ICUs) [188]. Besides, the positive influence of low dose Tocilizumab (400 mg) on 85 patients with COVID-19 pneumonia and severe respiratory failure not only supported the clinical effectiveness as well as the safety of this dosage but also proposed that early use in severe cases might promote clinical course and outcomes [189]. However, one new case report introduced two patients with COVID-19-related cytokine storm who received Tocilizumab therapy but later progressed to sHLH and even viral myocarditis, which cast doubt on the safety of Tocilizumab [190]. Even though the National Health Commission of China adopted Tocilizumab in COVID-19 therapy recommendations and existing studies have yielded initial conclusions regarding the safety and effectiveness of anti-cytokine interventions, the finding could be biased by a small sample, the effects of other therapies and the absence of randomized groups. To offer more solid evidence for the use of Tocilizumab, many institutions have launched clinical trials to evaluate its efficacy and safety. In addition to 24 registered clinical trials on Tocilizumab, the use of other anti-cytokine agents in clinic practice such as IL-6 receptor inhibitors (sarilumab), IL-6 inhibitors (siltuximab, clazakizumab, sirukumab), anti-Interleukin-8(BMS-986253), inhibitors of IL-1ra(Anakinra) are now being tested. Moreover, in addition to the clinical use, more proof is required to address the diagnostic criteria of cytokine storm, the disease severity grade, and biomarkers such as cytokine measurement to guide appropriate timing for treatment and predict prognosis [172].

### JAK2 inhibitors

Various kinds of cytokines assume leading roles in the development of immune response by binding cellular receptors from different families and subsequently activating downstream cascade reaction. In particular, cytokine showing high affinity with type I and type II cytokine receptors are effective modulatory agents in immune-related disorders. They can further attach to the JAK-related signaling transduction to mediate inflammatory reaction [191].

The four members in the JAK family, JAK1, JAK2, JAK3, and tyrosine kinase-2 could either individually interact with cytokine receptors or selectively form a group with the other three members to exert such function, such as the combination of JAK1 and JAK2 in IFN-γ-relevant signaling transduction [192, 193]. Therefore, the selective inhibitors of JAK have attracted great attention for their use in the inflammation-driven diseases including rheumatoid arthritis (RA), inflammatory bowel disease, and dermatological conditions, even in a few new clinical indications such as COVID-19 [194, 195]. As the potential receptor of 2019-nCov [196–198], ACE2 failed to act as the natural protector of the lung after the virus attack and led to acute lung injury [199]. The high level of inflammatory cytokines might further downregulate the ACE2 and foster the progression of COVID-19 [194, 200]. Therefore, blocking the downstream signal transduction of these inflammatory cytokines might alleviate the negative effects of excessive inflammatory response. Besides, JAK inhibitor of high affinity with AP2-associated protein kinase 1 (AAK1), the regulator mediating the endocytosis of 2019-nCoV, could also be a highly efficient contributor to disrupting the invasion of the virus [201].

In terms of the affinity with AAK1 and the safety of drug dosage, Baricitinib, a novel selective inhibitor of JAK1 and JAK2 [202], is recommended to be a candidate in the COVID-19 treatment [19]. One open-label design in Italy included 12 patients with moderate COVID-19 pneumonia who received a 2-week combined therapy of ritonavir-lopinavir and Baricitinib. Notably, the therapy improved respiratory function and laboratory parameters without adverse effects on the cardiovascular and hematologic system or infection [203]. Several clinical trials have been adopted to justify the safety and effectiveness of Baricitinib in clinical practice (NCT04320277, NCT04321993, NCT04340232, NCT04346147, NCT04345289, NCT04358614, NCT04373044, NCT04393051, NCT04399798, NCT04362943). Nevertheless, some studies challenged clinical use in specific indications including reduction of lymphocyte, elevations of creatine kinase, elderly patients, and secondary infections [204], all of which are common in severe and critical patients. Meanwhile, the interference with JAKs signaling transduction also dampens the role of α- and β-IFN in antiviral response since, as mentioned before, the delayed IFN activation might hinder the efficacy of the virus clearance and worsen the lung inflammation [205].

In addition to Baricitinib, clinical trials focus on other JAK inhibitors such as ruxolitinib (NCT04348695, NCT04361903, NCT04331665, NCT04338958, NCT04337359, NCT04374149, NCT04334044, NCT04377620, NCT04362137, NCT04366232, NCT04355793, NCT04359290, NCT04348071) and tofacitinib (NCT04390061, NCT04332042) have also been initiated to explore the potential effective drugs.

### Convalescent plasma therapy

Convalescent plasma therapy refers to the collection of plasma from convalescent patients with protective antibodies and the transfusion of convalescent plasma to recipients in case of the occurrence of possible lethal complications [206]. In addition to the antibodies inhibiting virus replication, other derivative components in plasma such as anti-inflammatory cytokines could modulate the immune response by blocking complement, which, in particular, might contribute to the disruption of the cytokines storms in COVID-19 [207, 339]. According to the dynamic characteristics of antibodies in immune response among COVID-19 patients, the seroconversion time of IgM and IgG is about 13 days after the symptom onset [208] while the viral load reached the peak within 12 days [209]. Therefore, given the temporary absence of vaccines available for COVID-19, passive immunotherapy could defend against SARS-CoV-2 until the establishment of effective immune response for specific pathogen. Preclinical evidence has shown that SARS-CoV-2 could induce both cellular and humoral immunity to protect against re-exposure in rhesus macaques [210]. Successful treatment in previous viral pandemics such as hemorrhagic fevers (Ebola) [211], influenza(H1N1 and H5N1) [212–216], and other coronaviruses also implies the promising future of convalescent plasma therapy in COVID-19 [217, 218]. One research in China included 5 critically ill patients with ARDS who received convalescent plasma treatment (titer of SARS-CoV-2-specific antibody> 1:1000; titer of neutralizing antibody> 1:40) from the 10th day to the 22nd day after admission and found that four out of these (4/5,80%) patients showed improved respiratory, negative viral load, increasing specific antibodies and neutralizing antibodies [219]. To test the effectiveness and safety of immunoglobulin therapy, Duan et.al designed a prospective study that provided combined therapy of antiviral drug and convalescent plasma (titer of neutralizing antibody> 1:640 derived from recovered patients for 10 confirmed patients). Apart from significantly improved clinical symptoms and laboratory parameters such as increasing lymphocyte counts and decreasing CRP levels, no detection of virus or adverse effects were observed within 3 days after the initiation of the trial [220]. Nevertheless, despite the approval from FDA of using convalescent plasma in Critically Ill Patients [221], there might be potential hazards we have not been fully aware of, such as the high risk of antibody-dependent enhancement [222], since no large scale RCTs or registered clinical trials weighing the therapeutic benefits and potential risks of convalescent plasma have yielded convincing results [223]. Besides, the acquisition of robust humoral response, management and administration of convalescent plasma, transfusion reactions and reinfection could also be barriers to the clinical use of immunoglobulin therapy [224].

### Chloroquine (CQ) and hydroxychloroquine (HCQ)

Sharing similar chemical functions and therapeutic mechanisms, both CQ and HCQ are primarily anti antimalarial drugs but recently well recognized for its benefits in many various diseases, especially in rheumatic and skin disorders such as RA [225], systemic lupus erythematosus [226] and antiphospholipid syndrome [227]. The possible mechanism for its immunomodulatory and anti-inflammatory effects mainly include:(1) increasing the PH to inhibit the lysosomal activity and therefore dampening the lysosome-mediated antigen processing [228–230] (2) inhibiting Toll-like receptor (TLR) signaling [231, 232] (3) interfering type I IFN response by modulating the activity of cyclic GMP-AMP (cGAMP) synthase [233] (4). reducing the production of proinflammatory cytokines of macrophage [234–236]. CQ and HCQ also possess antiviral properties by interfering with the different stages of virus replication:(1) suppressing glycosylation of ACE2, the target shared by both SARS-CoV-1 and SARS-CoV-2 [198] (2) destruction of the PH-dependent virus-endosome fusion to inhibit the release of virus RNA [237] (3) impairing proteolytic processes and glycosylation to interfere with the posttranscriptional modification [238, 239]. However, while some studies have reported the antiviral activity in HIV [240, 241], Zika virus [242], influenza virus [243], MERS-CoV [74], and SARS-CoV-1 [244], opposing opinions always exist arguing the uncertain role of CQ and HCQ in vivo studies. In mice and cat models, CQ did not present a significant antiviral effect though it might participate in the modulation of the immune response [235, 245].

Recent experiments in vitro also demonstrated that both HCQ and CQ could effectively block the virus replication in Vero cells infected with SARS-CoV-2 at low concentration [87, 119, 246]. But few in vivo studies have yielded meaningful data to support the conclusion. In one research, while patients receiving either azithromycin or hydroxychloroquine had a lower rate of virus clearance, those following combined treatment showed a negative nasopharyngeal swab after 6 days [247]. Another research aimed to replicate such amazing therapeutic benefits by executing the same design in 11 patients but failed [248]. According to a meta-analysis [249] reviewing the current seven studies evaluating the antiviral property in patents, five gave support to the efficacy of HCQ or CQ while the other two did not. In one study, a multicenter, open-label, randomized controlled clinical trial including 150 patients, 75 patients received 1200 mg HCQ treatment for 3 days, and then followed a daily maintenance dose of 800 mg in addition to standard of care assigned to the control group. While 56 patients in the control group displayed positive conversion, 53 patients in the experiment group showed negative conversion within 28 days. Besides, adverse events were higher in the hydroxychloroquine treatment group (21/70,30% versus 7/80,9%). Therefore, there are no significant beneficial effects of HCQ treatment on negative conversion but a higher chance to suffer adverse events [250]. A similar observational study acquired a similar conclusion that neither the mortality nor the composite endpoint of intubation was related to the hydroxychloroquine treatment [251]. The latest study comparing 1438 COVID-19 patients who received HCQ and Azithromycin respectively, or the combination of the two with the control group indicated that no significant difference of the in-hospital death among the four treatment groups was observed but the possibility of cardiac arrest significantly increased in patients following combined treatment [252]. Registered clinical trials were recruiting volunteers to test the efficacy and safety of CQ and HCQ at different dosages in prevention and treatment combined with other drugs or without.

Although these studies have primarily provided some initial evidence for the use in the clinic, the data available now could not sufficiently warrant the application of CQ or HCQ in clinical practice nor against them. Therefore, even if some guidelines recommend an emergent use of HCQ or CQ, results of well-designed clinical trials are urgently needed to verify the feasibility of HCQ treatment on COVID-19 patients as well as instructions for proper administration to avoid severe adverse effects such as gastrointestinal symptoms, neurologic side effects, cardiomyopathy and conduction disturbances [253].

### The IFN family

Apart from the antiviral property described above, IFN also acts as a modulatory agent in both innate and adaptive response. IFN contributes to the enhancement of the cytotoxic activity of natural killer cells [254, 255], growth and differentiation of dendritic cells (DC) [256], and regulation of T-cell and B-cell response [257, 258]. In addition to the traditional use in HBV and HCV [259], IFN also proves to be effective in treating SARS-CoV-related infection. For instance, the combined therapy of IFN-1 and glucocorticoid could improve oxygen saturation and radiographic lung abnormalities in SARS patients [260]. For COVID-19, combined therapy of IFN and other antiviral drugs have achieved some initial results. Among patients who received combined treatment of IFN-α + lopinavir/ritonavir or IFN-α + lopinavir/ritonavir + ribavirin, the mRNA clearance rate was in positive correlation with hospitalization length, which might imply the benefits of combined treatment [151]. Ongoing clinic trials may further reveal the promising future of IFN in COVID-19 treatment. Some studies also discussed the possible beneficial effect of interferon lambda on virus load decrease as well as suppression of hyperactive inflammation response [261, 262]. Meanwhile, among 4 completed clinical trials (NCT04343768, NCT04389645, NCT04291729, NCT04276688), an open-Label, randomized, phase 2 trial (NCT04276688) published their result that early combined therapy of IFNβ-1b, Lopinavir-Ritonavir, and Ribavirin improved clinic course, accelerated viral shedding and shortened hospitalization length in patients with mild symptoms, which implied the efficacy of interferon beta-1b as a backbone in antiviral therapy [130].

### Intravenous immunoglobulin (IVIG)

Extracted IgG from thousands of healthy plasma donors, Intravenous immunoglobulin preparations contain both immune antibodies for replacement therapy and passive immunity and physiologic autoantibodies for immunomodulation [263]. While the efficacy of IVIG has been verified in immune-related and neurological disorders, it’s hard to develop a clear systematic understanding of the mechanism. The potential therapeutic benefit mainly depends on the two functional domains of IgG, F (ab′)2 and Fc and fragment, the function of which including cellular receptor blockade, suppression of Fc gamma receptors expression and activation, a saturation of the neonatal Fc receptor, regulation of cytokines, complements and immunocytes, Fc-dependent immunomodulatory pathway [207, 264, 265].

While according to a systematic review [134], two studies focusing on the effectiveness of IVIG during SARS were inconclusive [266, 267], preclinical evidence primarily demonstrated that antibodies in intravenous immunoglobulins could crossreact against SARS-CoV-2 [268]. Xie et.al first reported their retrospective study of 58 severe or critical patients who receive IVIG as adjuvant treatment within 48 h or more than 48 h after admission to ICUs and found that IVIG treatment within 48 h could improve their ventilation function, reduce hospitalization length and 28-day mortality [269]. Also, two case reports claimed successful IVIG treatment in COVID-19 patients [270, 271]. While these positive results may provide preliminary evidence for IVIG treatment in COVID patients, more large scale prospective randomized controlled trials would further warrant the use. Following clinical trials (NCT04383548, NCT04400058, NCT04261426, NCT04264858, NCT04350580) plan to test the therapeutic effect of various immunoglobulin on COVID-19 patients and provide more convincing proof regarding the use.

### Summary of immunotherapy

Inflammation is conducive to the elimination of pathogens in the immune response. However, in the severe case of COVID-19, SARS-CoV-2 could induce an excessive immune response and cytokine storm, which finally leads to lethal complications such as ARDS and multiple-organ dysfunction. While the antiviral drugs and symptomatic therapy remain the major treatment, immunomodulatory therapy including corticosteroids, anti-cytokine agents, JAK inhibitors, chloroquine, hydroxychloroquine, convalescent plasma, IVIG and IFN, could alleviate the local or systemic inflammation injury and further prevent the progress of COVID-19.

However, the major issue regarding the use of immunomodulatory agents is the proper administration timing, safe and effective dosage, and clinical indications. Immunomodulatory agents could sometimes inhibit the immune response and engender secondary infection or delay the pathogen clearance. Besides, since severe cases tend to suffer hyper inflammation and soon deterioration, the onset of the anti-inflammation therapy is crucial for reversing the conditions. Finally, in addition to the perplexing mechanism of these therapies, conflicting evidence regarding the effectiveness further refrain clinic use.

Observation studies currently available subject to limited sample numbers, short follow-up duration and different administration plan fails to provide convincing conclusions supporting the wide application in larger groups of patients. Besides, no clinical trials or literature focusing on the therapeutic effects of immunomodulatory agents on the COVID-19 patients have achieved an agreement so far. Therefore, without official guidance, the clinicians must caution against the use of immune therapy and give personal administration based on the overall evaluation of the patient’s disease condition, for instance, the appropriate medication time and dosage.

## Other therapies

### Mesenchymal stem cells

Mesenchymal stem cells (MSCs) are generally prepared from MSCs isolated from unrelated donor bone marrow and amplified in culture [272, 273]. MSCs have now been shown to have immunomodulatory effects when administered via intravenous infusion [274]. The anti-inflammatory effect is achieved by down-regulating the production of pro-inflammatory cytokines and increasing the production of paracrine and anti-inflammatory cytokines for tissue repair. What’ more, MSCs can recruit natural anti-inflammatory cells into relevant tissues to fight the inflammatory processes associated with many diseases [78]. Preclinical evidence suggests that MSCs have the ability to restore endothelial cell permeability and reduce inflammatory infiltration. Although the immunomodulatory role of MSCs has been demonstrated on avian influenza viruses, their role in COVID-19 is still being evaluated [188]. Currently, There were some clinical trials to evaluate the efficacy of MSCs which were from the umbilical cord and pulp (NCT04293692, NCT04269525, NCT04288102, NCT04302519) [275–277].

Mesoblast LTD has been approved by the FDA for a new drug clinical trial application for its allogeneic cell therapy Ryoncil (remestemcel-L). Administration via intravenous infusion for the treatment of acute respiratory distress syndrome in patients with COVID-19. In the study, which was conducted in over 1100 patients, the safety and therapeutic efficacy of intravenous administration of remestemcel-L have been evaluated. These patients had a variety of inflammatory diseases, including elderly patients with lung disease, adult and pediatric steroid-refractory acute graft-versus-host disease, chronic graft-versus-host disease, biologically agent-refractory Crohn’s disease, hypoxic-ischemic encephalopathy, and herpetic epidermolysis [275, 276, 278]. Studies have shown that the cytokine storm process produced by COVID-19 is similar to aGVHD. In addition, a study of 60 patients with chronic obstructive pulmonary disease showed that remestemcel-L had the obvious ability to improve respiratory function in patients with the same elevated inflammatory biomarkers that were also observed in patients with COVID-19 ARDS [279]. These provide a theoretical basis for the treatment of COVID-19 by remestemcel-L.

Leng et al. showed that homozygous ACE2-MSC transplantation was effective in improving the prognosis of COVID-19 [280]. In seven patients with COVID-19 who received a single intravenous MSC graft, the results showed that MSC cured or significantly improved lung function in these 7 patients within 14 days of transplantation, with no significant adverse effects. In addition, MSCs are known to exhibit significant immunomodulatory functions. On day 4 after transplantation, C-reactive protein levels in critically ill patients decreased from a maximum of 191.0 g/L to 13.6 g/L, and absolute lymphocyte counts increased to 0.58 × 10^9^, indicating rapid remission of inflammation, significant improvement in lymphocyte reduction, and return to normal biochemical indicators of liver and heart function [280].

### Herbal medicine

In previous treatment experience, herbal medicine has a role in preventing SARS and H1N1 influenza. There are two common prescriptions: Yupingfeng Powder and Sangju Decoction. Some studies have shown that Yupingfeng Powder has antiviral, anti-inflammatory and immunomodulatory effects [281, 282]. The main use of the herb is for upper respiratory infections, antibacterial and antiviral effects, improving the immune system of the upper respiratory tract mucous membrane.

In the treatment of H1N1 influenza is, commonly used Qingjie Fanggan Granule, Ganmao Qingre Granule, Kangbingdu Oral Liquid. In a meta-analysis, the infection rate was significantly lower in patients taking these herbal formulations than in the control group. Relative risk (RR) 0.36, 95% confidence Interval 0.24–0.52, *P* < 0.01. A total of 54 different herbs are currently in use in herbal remedies [196, 283–285]. Astragalus and licorice are used the most. However, the mechanism of herbal treatment is currently unclear and strong evidence is lacking. The formulas used vary from province to province. But the safety of herbal medicine use is of great concern. The formulas used are different for different ages, so be sure to use them under the guidance of your doctor. There should be more retrospective, RCT studies in the future to evaluate the preventive role of herbs in COVID-19.

## Vaccines

Because of the enormous impact of COVID-19 on human health, research institutes in various countries have been developing vaccines. The first candidate 2019 coronavirus vaccine entered human clinical testing at an unprecedented rate on March 16, 2020. Evaluation of the next generation of vaccine technology platforms is also being promoted through new models [286–288].

### mRNA vaccine

The mRNA vaccine is the delivery of mRNA to cells that express the protein that produces it, thereby expanding the immunity of the organism [288]. It does not require any nuclear localization signal, transcription, and integration into the genome is not possible, which avoids any possible therapeutic mutations [289, 290]. There are two main types of mRNA vaccines available, those that are self-amplifying and those that are non-replicating mRNA [291]. Self-amplifying mRNA vaccines are usually based on the genome of the genus alphavirus, where the gene encoding the RNA replication mechanism is intact and the structural protein-coding gene of the protovirus is replaced with mRNA encoding the antigenic protein [292, 293]. Non-replicating mRNA vaccines are in vitro transcribed sections of complete mRNA encoding antigenic proteins, including 5′ and 3′ untranslated regions, and poly(A) tail to stabilize the mRNA and promote transcription [294, 295]. The mRNA vaccine is synthesized by in vitro transcription techniques using plasmid DNA or other DNA fragments containing the open reading frame of the target protein as a template [296].

Since mRNAs contain cap structures at the 5′ end and Poly(A) at the 3′ end, the addition of these components is generally required after in vitro transcription of the synthesized mRNA. There are also many current studies on the synthesis of mRNA vaccines in vitro by chemical modification. And for mRNA vaccine delivery, it can be done by electroporation, liposome nanoparticle delivery system, polymer delivery system [297–300]. For example, Nucleoside-modified mRNA greatly improves mRNA stability and can regulate the half-life of mRNA drugs in vivo; liposomal nanoparticles can envelope mRNA, further improving stability and also efficiently complete intracellular delivery of mRNA [296, 301].

The first candidate COVID-19 vaccine was mRNA-1273 developed by Moderna. After Chinese scholars shared the gene sequence of the SARS-CoV-2 on January 11, 2020, NIH and Moderna began development of the mRNA-1273 vaccine with funding from The Coalition for Epidemic Preparedness Innovations (CEPI). On February 7, production of the first clinical batch of the vaccine was completed, and on March 4, a Phase I clinical trial was approved by the FDA. This experiment provided important data on the safety and immunogenicity of mRNA-1273 by recruiting 45 healthy adult volunteers aged 18 to 55 years.

The clinical phase I trial conducted by Moderna had three dose groups, 25μg, 100μg, 250μg, and expanded the six groups in the older and the elder. On May 7, the FDA approved the study for a Phase II clinical trial. A third phase of the study is planned for early summer. The platform used for this vaccine is mRNA. In past studies, the safety of Phase I clinical trial species of five other respiratory viruses (two pandemic influenza viruses, RSV, hMPV, and PIV3) has been proved [302, 303]. mRNA is an information molecule, and Moderna used the sequence of the virus to design messenger RNA vaccines, rather than studying the virus itself. mRNA platforms have significant advantages in terms of speed and efficiency. mRNA can span basic science, manufacturing, and clinical development. After verifying the safety and efficacy of mRNA-1273, it will be put into mass production. On May 18, data from the Phase I clinical trial published on Moderna’s webpage showed that, after two doses, all participants in the 25 μg and 100 μg dose cohorts evaluated to date had seroconversion rates that met or exceeded the levels of conjugated antibodies in their recovery serum. In the 25 μg and 100 μg dose cohorts, mRNA-1273 elicited neutralizing antibody titers in all eight initial participants, meeting or exceeding the neutralizing antibody titers typically seen in recovery serum. mRNA-1273 was overall safe and well tolerated. The only grade 3 adverse event that occurred in the 25 μg and 100 μg dose cohorts was a grade 3 erythema around the injection site in a participant in the 100 μg dose group. By far, the most notable adverse events occurred at the 250 μg dose level, with three participants experiencing grade 3 systemic symptoms only after the second dose. All adverse events are transient and can resolve themselves. No grade 4 adverse events or serious adverse events are reported [304].

### DNA vaccine

DNA vaccines are delivered into the body and are taken up by surrounding tissue cells (e.g., myocytes), antigen-presenting cells (APCs) or other inflammatory cells [305]. Plasmid DNA molecules ingested by tissue cells such as myocytes are then transcribed into mRNA in the nucleus, which is then moved into the cytoplasm for translation into antigenic protein molecules [306]. The antigenic protein molecules released by the cells into the tissue interstitium are captured by APCs and processed into antigen-peptide delivery to T cells, initiating an immune response [307]. APCs from peripheral lymphatic organs also directly uptake nucleic acid vaccines, express antigens and deliver them to T cells, triggering an immune response. Dendritic cells are the most important antigen-delivering cells in nucleic acid immunity, while B cells are not involved in the antigen-delivering process [308]. After triggering an immune response, the cytotoxic T-cell (CTL) response recognizes and kills myocytes expressing exogenous antigens, causing myocytes to lyse and release intracellular antigens, which APC obtains directly from the injection site to initiate the subsequent immune response. The combination of several pathways allows the DNA vaccine to stimulate T lymphocytes via the histocompatibility complexes MHC I and MHC II, and to activate B lymphocytes. Tissue cells such as myocytes may act as storage plasmids and regular release during the immune process [25, 309].

Inovio had developed a Phase 2 vaccine for Middle Eastern respiratory syndrome-related coronavirus, has designed INO-4800 using the DNA medicines platform. INO-4800 matches the DNA sequence of the virus precisely. On April 20th, Clinical Phase I trial has been approved by the FDA. DNA medicine consists of optimized DNA plasmids or recombined by computer sequencing techniques and designed to produce specific immune responses in the human body. Inovio’s DNA drugs use Inovio’s patented handheld smart device, CELLECTRA®, to deliver optimized plasmids directly into cells by intramuscular or intradermal injections.

CELLECTRA uses a short electrical pulse to reversibly opens small pores in the cell to allow plasmids to enter by using a short electrical pulse to reversibly. Once in the cell, the plasmids begin to replicate, thus reinforcing natural response mechanisms. The use of CELLECTRA device makes sure that the DNA drug enters cells directly, where it can immediately initiate an immune response. Inovio’s DNA drugs do not interfere with or alter a person’s DNA in any way. The advantages of Inovio’s DNA drug platform are fast development and production of DNA drugs, good product stability, no refrigeration for storage and transport, strong immune response, safety and tolerability.

### Recombination vaccine

The main mechanism of recombinant COVID-19 vaccines (adenovirus vectors) is the use of genetic engineering techniques to introduce and express genes encoding pathogenic protective antigens into adenovirus vaccines [310]. The first step is to select the highly characteristic protein structures on the surface of the pathogenic virus, that is, these protein structures stimulate the immune system to produce antibodies. For coronaviruses, the protrusion on the surface of the viral shell (S protein) is a target protein. Next, find the gene that encodes the S protein [311, 312]. For DNA viruses, the corresponding DNA fragment can be found directly; for RNA viruses, the corresponding RNA has to be found and translated into DNA fragments [313, 314]. What’ more, the encoded genes are fused into the DNA of the adenovirus and allowed to enter the human cell via the adenovirus as a vector. Finally, these coding genes synthesize some of the characteristic proteins of the pathogenic virus in the body, which induces strong humoral and cellular immunity and induces the body to produce specific antibodies, which are people’s immunity against the pathogenic virus [315].

### Ad5-nCoV

Ad5-nCoV, a recombinant vaccine (adenovirus vector type 5) studied by the Beijing Institute of Biotechnology and three other Chinese research institutions with support from CanSino Biologics Inc. [316]. A clinical phase I trial (NCT04313127) has already started on March 15, 2020, which is a single-center, open-label, dose-escalating, phase I clinical trial in a healthy population aged 18 to 60 years to assess the safety, adverse effects and immunogenicity of a novel recombinant coronavirus vaccine. One hundred eight volunteers were assigned to three groups and received either an intramuscular injection of the experimental vaccine in the deltoid muscle or a placebo. The experimental group was divided into high school and low three dose groups, and the estimated completion time of this clinical trial is December 2020.

The study conducted by Wei Chen et al. was published in The Lancet on May 22nd, and the safety, tolerability and immunogenicity of Ad5-nCoV were reported [317]. The main findings so far show that Ad5-nCoV is safe, well-tolerated in humans, and able to cause the immune response of immune system to COVID-19. Further trials will be required to assess whether the vaccine is effective in preventing neo-coronavirus infection. In the article, it was reported that within 7 days of Ad5-nCoV vaccination, 30 people in the low-dose group (83%), 30 people in the medium-dose group, 30 People (83%) and 27 people (75%) in the high dose group experienced at least one adverse effect. These adverse reactions included more than half (54%, 58/108) of the vaccines experiencing mild pain at the injection site. Fever (46%, 50/108), fatigue (44%, 47/108), headache (39%, 42/108) and muscle pain (17%, 18/108). The results showed that each dose of vaccine was well tolerated and no serious adverse reactions were reported within 28 days after inoculation. Most adverse events were mild or moderate.

Reports of immunogenicity of Ad5-nCoV showed that within 14 days of vaccination, a certain level of immune response was triggered and antibodies were produced in the vaccines. The specific ratios were 16/36, 44%, in the low-dose group; 18/36, 50%, in the medium-dose group; 22/36, 61%, in the high-dose group. Antibodies were produced at detectable levels in some subjects; the vaccine also triggered T-cell response. Twenty-eight days after vaccination, T-cell responses, or detectable levels of neutralizing antibodies, were present in the majority of vaccines. The specific ratios were: 28/36, 78% in the low-dose group, 33/36, 92% in the medium-dose group, 36/36, 100% in the high-dose group. The researchers also found that if pre-existing immunity to adenovirus Ad5 existed in the subjects, the vaccine could be Weakening, such as reduced peak levels of immune responses and shortened persistence of immune responses.

Only 108 volunteers were involved in this study, and the short duration of the trial, as well as the lack of randomized controls, made it difficult to detect Adverse events, or the discovery of limitations in the protective power of vaccines. A phase II, randomized, double-blind, controlled clinical trial involving 500 volunteers is currently underway in Wuhan to see if the results of this phase I clinical trial can be replicated and if adverse events occur within 6 months of vaccination. What’ more, the population who are 60 years of age was also involved as subjects, for the first time.

### ChAdOx1 nCoV-19

ChAdOx1 nCoV-19, developed at the University of Oxford, consists of a non-replicating adenovirus vector and the S protein gene sequence of SARS-CoV-2 and is in Phase I/II clinical trials (NCT04324606). Adenovirus does not replicate in the host, making it relatively safe in children and individuals with underlying diseases. In addition, based on the carrier of adenovirus has extensive organization orientation, including respiratory and gastrointestinal epithelium, both express the ACE of SARS-CoV-2 main parts of the receptor. Should always consider the carrier gene, however, rather than the possibility of genetically modified dominant immunogenicity [318].

According to the current results of animal experiments on ChAdOx1 nCoV-19 in rhesus monkeys, ChAdOx1 nCoV-19 vaccine does not prevent macaque monkeys from contracting the virus, nor does it prevent animals from spreading the infection to other animals. In this study, six rhesus macaques were vaccinated with the ChAdOx1 nCoV-19 vaccine, and after 28 days were exposed to the SARS-CoV-2 virus. The researchers also compared them to three unvaccinated monkeys. As determined by the recovery of viral genomic RNA in nasal secretions, the researchers determined that all six macaques that were vaccinated with the vaccine were infected with the COVID-19. Compared with unvaccinated animals, the amount of viral RNA detected from this site in vaccinated rhesus monkeys had no difference.

### COVID-19 synthetic Minigene vaccine

DC and CTL cells play a key role in viral clearance during the immune process, so it is important to induce vaccines that produce strong, long-lasting, cross-T cell responses [319–322]. This minigene can express a segment of amino acid residue peptide through viral infection or the synthesis of a minigene. Infected cells can sensitize immune cells and stimulate T-cell activity [323, 324].

Based on a detailed analysis of the viral genome and the finding for latent immunogenic targets, a synthetic mini-protein based on the conserved structural domains of viral structural proteins and multiprotein proteases was synthesized by the Shenzhen Geno-Immune Medical Institute. COVID-19 infection is mediated by binding of the spike protein to the ACE2 receptor and viral replication depends on the molecular mechanism of all these viral proteins. This experiment intends to use the efficient lentiviral vector system (NHP/TYF) to develop and test a novel COVID-19 mini-genome based on a variety of viral genes, express viral proteins and immunoregulatory genes, modify DCs, and activate T cells [325]. In this study (NCT04276896), the safety and efficacy of the LV vaccine (LV- SMENP) will be investigated.

### Summary of vaccines

A feature of the COVID-19 vaccine development field is the search for a wide range of platforms, including nucleic acids (DNA and RNA), virus-like particles, peptides, viral vectors (replicated and non-replicated), recombinant proteins, attenuated live viruses and inactivated virus methods [326, 327]. Although many of the methods are not vectors used in conventional vaccine studies, they may work well for specific populations (such as elderly, pregnant women, children) [328–330]. For some platforms, adjuvants can enhance immunogenicity and make low doses feasible, allowing more people to be vaccinated without compromising protection [331, 332]. Ten researchers have already started this study. Common adjuvants are AS03, MF59 and CpG 1018 which are made by GlaxoSmithKline, Seqirus and Dynavax [3, 333–335].

The COVID vaccine is expected to be in use by early 2021, which could reduce the 10-year lead time for a conventional vaccine to be successfully developed at a time when a lot of manpower and resources are currently being invested [336, 337]. Current accelerated vaccine development through parallel and adaptive development phases, innovative regulatory processes and expanded manufacturing capabilities [25, 338].

## Conclusion

**Fig. 2:**
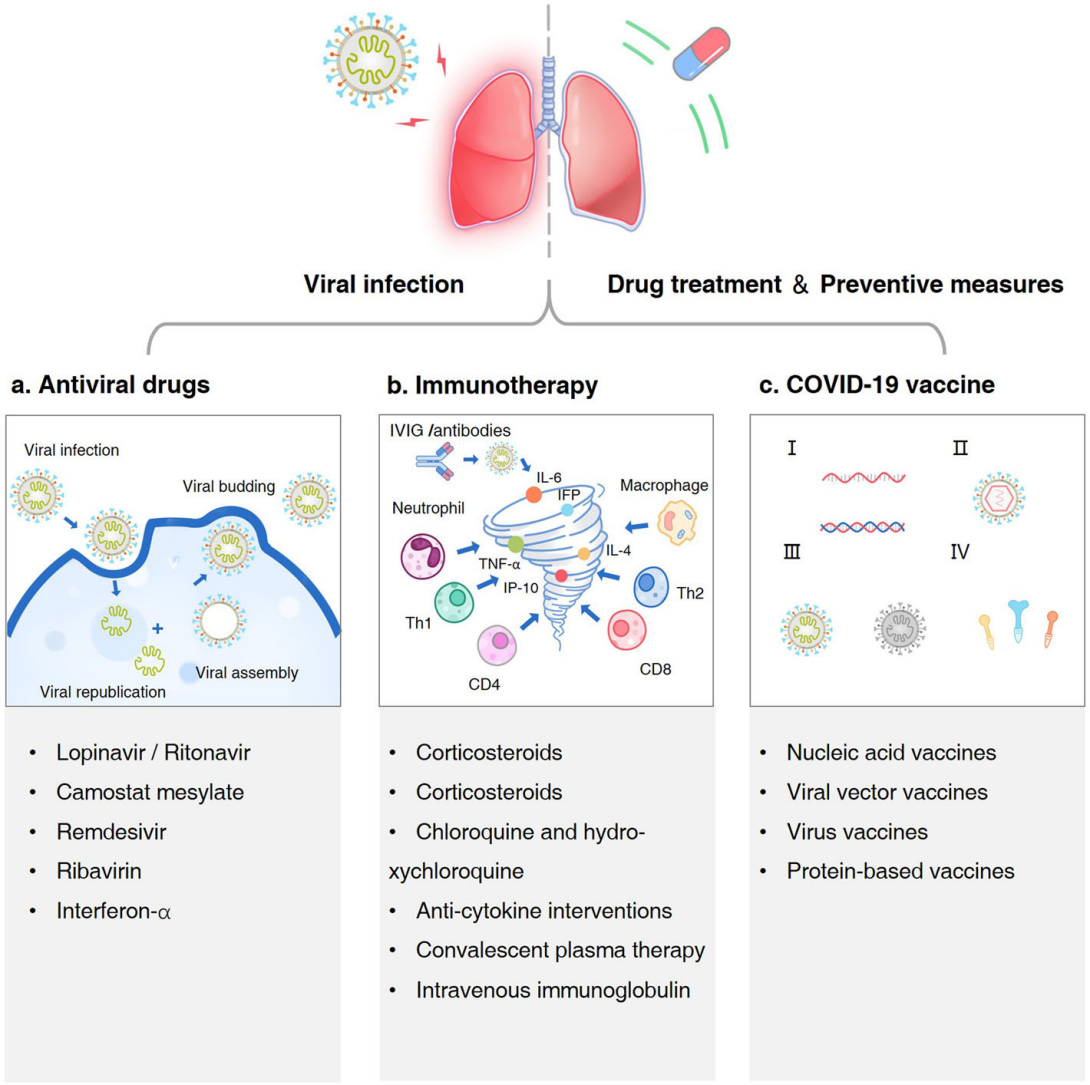
A brief overview of drug treatment and preventive measures. Antiviral drugs: Lopinavir, ritonavir, and camostat mesylate are two protease inhibitors, and the pesticide effect of lopinavir improves when combined with ritonavir usage. Remdesivir, a nucleotide analogue, can inhibit the proliferation of the virus by targeting the RNA polymerase. It can act as a nucleotide analogue, an immunomodulator, and promote RNA degradation. Interferon-α can inhibit the virus by multiple immune pathways. Immunotherapy: Corticosteroids prevent the overactive immune response as well as the progression of pulmonary fibrosis. Chloroquine and hydroxychloroquine act as an immunomodulatory agent by dampening the lysosome-mediated antigen processing, inhibiting Toll-like receptor signaling, interfering with type I IFN response, and reducing the production of proinflammatory cytokines. Anti-cytokine interventions contribute to suppressing the lung injury caused by the cytokine storm. Convalescent plasma therapy and Intravenous immunoglobulin can enhance passive immune response as well as provide other derivative components in plasma to modulate immunity. COVID-19 vaccine: Nucieic acid vaccines include DNA and RNA. The platform of viral vector vaccines is adenovirus type 5 vector. Virus vaccines have codon deoptimized live attenuated and inactivated SARS-Cov + Alum. Protein-based vaccines are full-length recombinant SARS CoV-2 glycoprotein and nanoparticle vaccine adjuvanted with Matrix M

Under the currently emergent state of global health conditions resulting from COVID-19, effective therapies are desirably needed. The review introduced possible therapeutical treatments and their underlying mechanism against the SARS-CoV-2 infection (Fig. 2). Research on the repurpose of antiviral drugs has acquired some preliminary results proving their efficacy in forestalling virus reproduction at different stages or blocking specific targets including Lopinavir/ritonavir, Ribavirin and Remdesivir. Furthermore, given the hyper inflammation response mediated by the dysregulation of the immune system, the anti-inflammation or immunomodulatory agents are expected to play roles in preventing deterioration. However, despite the theoretical feasibility, the proper administration timing, safe and effective dosage, and clinical indications remains to be undetermined and therefore require a cautious assessment of the patient’s health conditions before administration to avoid potential adverse effects. Besides, we also updated the newest progress for both other adjuvant treatments such as herbal therapies or mesenchymal stem cell therapies and the vaccine under different stages of clinical trials which soon will be in use to alleviate the heavy burden of increasingly worsening global health conditions and economic plight. Nevertheless, due to the current absence of conclusive proof to support the recommendation for specific therapies or vaccines in the clinical practice, further researches and large scales clinical trials might serve to equip clinicians with more valid information regarding the efficacy and pharmacokinetics of specific therapy of vaccines and therefore optimize their clinical strategies.

## Data Availability

Not applicable.

## Abbreviations

AAK1: AP2-associated protein kinase 1
ACE2: Angiotensin-converting enzyme 2
ADAR: RNA-specific adenosine deaminase
APCs: Antigen-presenting cells
ARDS: Acute respiratory distress syndrome
CM: Camostat mesylate
COVID-19: Coronavirus disease 2019
CQ: Chloroquine
CRP: C-reactive protein
CT: Computed tomography
CTD: C-terminal domain
CTL: Cytotoxic T-cell
DCs: Dendritic cells
EC50: 50% effective concentration
FDA: Food and Drug Administration
hACE2: Human ACE2
HCQ: Hydroxychloroquine
HIV: Human immunodeficiency virus
ICUs: Intensive care units
IFN: Interferon
IL-6: Interleukin-6
IFN: Interferon
IVIG: Intravenous immunoglobulin
JAK: Janus kinase
LPV/r: Lopinavir/ritonavir
MERS: Middle East respiratory syndrome
MERS-CoV: Middle East respiratory syndrome coronavirus
MSCs: Mesenchymal stem cells
NF-κB: Nuclear factor-KappaB
OAS: Oligoadenylate synthetase
PKR: Double-stranded RNA-dependent protein kinase
RA: Rheumatoid arthritis
RCTs: Randomized controlled trials
RMP: Ribavirin monophosphate
RNase L: Ribonuclease L
RR: Relative risk
RT-PCR: Reverse transcriptase-polymerase chain reaction
RTV: Ritonavir
SARS: Severe acute respiratory syndrome
SARS-CoV: Severe acute respiratory syndrome coronavirus
SARS-CoV-2: Severe acute respiratory syndrome coronavirus 2
SB: Domain B of spike protein
TLR: Toll-like receptor
TMPRSS2: Transmembrane protease/ serine subfamily member 2
